# Development of a Clinical Prediction Model for Recurrent Anaphylaxis

**DOI:** 10.3390/jcm15113990

**Published:** 2026-05-22

**Authors:** Suwannee Uthaisangsook, Sagoontee Inkate, Susita Wangchiraniran

**Affiliations:** 1 Department of Pediatrics, Faculty of Medicine, Naresuan University, 99 Tha Pho, Mueang District, Phitsanulok 65000, Thailand; susitaw@nu.ac.th; 2 Department of Family Medicine, Faculty of Medicine, Naresuan University, 99 Tha Pho, Mueang District, Phitsanulok 65000, Thailand; sagoontee@gmail.com

**Keywords:** prediction model, predictive factors, recurrent anaphylaxis, risk factors, risk score

## Abstract

**Background/Objectives**: Preventing recurrent anaphylaxis is crucial for patient safety. This study aimed to identify predictive factors and develop a prediction model to estimate recurrence risk, thereby enhancing targeted preventive strategies. **Methods**: This prognostic prediction study used a retrospective observational cohort design, analyzing medical records from an anaphylaxis registry at Naresuan University Hospital, Phitsanulok, Thailand, between March 2011 and February 2021. We developed a prediction model using multivariable Cox proportional hazards regression analysis. Statistically significant and clinically relevant predictors were weighted into a risk score derived from hazard ratio regression coefficients. Model performance was evaluated using the area under the receiver operating characteristic curve (AuROC), calibration metrics, and decision curve analysis. **Results**: Over the 10-year period, 381 patients experienced 439 anaphylaxis episodes, including 58 recurrences (13.2%). The final model comprised six predictors: history of food, insect, and drug allergies; asthma; chest discomfort; and severe anaphylaxis. Corresponding risk scores were 4, 5, 5.5, 1, 2.5, and 1.5 points, respectively. Total scores ranged from 0 to 19.5 and were categorized into low (<3.0), moderate (3.0–9.0), and high (>9.0) risk groups. The high-risk group had a likelihood ratio positive (LHR+) of 4.65. The model demonstrated acceptable discrimination (AuROC 0.773 (95% CI: 0.714–0.832)) and good calibration. Bootstrap validation showed consistent performance (AuROC 0.773 (95% CI: 0.714–0.831)). Decision curve analysis indicated clinical utility across relevant threshold probabilities. **Conclusions**: This prediction model provides a simple, clinically applicable tool for estimating the risk of recurrent anaphylaxis and may support improved prevention and management strategies.

## 1. Introduction

Anaphylaxis is a severe and potentially fatal systemic allergic reaction that requires prompt recognition and urgent medical intervention to prevent life-threatening outcomes. The reported incidence of anaphylaxis ranges from 1 to 761 episodes per 100,000 person-years across both adult and pediatric populations worldwide, with an estimated lifetime prevalence of 0.3% to 5.1% [1,2,3]. Importantly, recurrent anaphylactic episodes are common, with reported recurrence rates ranging from 26.5% to 54% over follow-up periods of 1.5 to 24 years [4]. In pediatric populations, an annual recurrence rate of 17.6% has been documented [5]. Other studies have demonstrated substantial variability in recurrence rates, ranging from 6.6 to 90 episodes per 100 person-years in children [6] and from 1.4 to 57 events per 100 person-years across all age groups [7,8,9,10]. In Thailand, our recent study reported a recurrence rate of 4.1 events per 100 person-years [11].

Prevention of recurrent anaphylaxis is therefore of paramount importance. Identifying risk factors and predictive markers for recurrence enables clinicians and patients to implement proactive mitigation strategies. One observational cohort study identified a history of atopic dermatitis, oral pruritus, cough, and steroid therapy as significant risk factors associated with recurrence [7]. Other studies have highlighted additional risk factors, including a history of asthma, atopic dermatitis, food allergy, allergic rhinitis, age <18 years, and reaction triggered by food [5,7,9,10,12,13,14]. Our preceding research demonstrated that recurrent anaphylaxis is associated with prior food, drug, or insect allergies, alongside acute index presentations of chest discomfort and severe anaphylaxis [11].

Despite these established associations, investigations focusing on the development of predictive models for recurrent anaphylaxis remain limited. Therefore, this study aimed to identify predictive risk factors and develop a clinical prediction model capable of estimating the risk of recurrent anaphylactic episodes. The application of such a model may facilitate improved clinical decision making, enable closer monitoring of high-risk patients, and ultimately reduce the risk of recurrent or fatal anaphylaxis.

## 2. Materials and Methods

### 2.1. Study Design and Setting

This prognostic prediction study utilized a retrospective observational cohort design, analyzing data from an anaphylaxis registry between March 2011 and February 2021 at Naresuan University Hospital, a tertiary care center in lower northern Thailand [11,15]. Medical records of patients experiencing anaphylaxis were reviewed and stratified into recurrent and non-recurrent cohorts.

### 2.2. Study Population and Data Collection

Patients with anaphylaxis were identified from medical records using ICD-10 codes, including T78.0 (anaphylactic shock due to an adverse food reaction), T78.2 (unspecified anaphylactic shock), T80.5 (anaphylactic shock due to serum), and T88.6 (anaphylactic shock due to an adverse drug effect). The diagnosis of anaphylaxis was subsequently confirmed according to the clinical criteria established by the second U.S. National Institute of Allergy and Infectious Diseases/Food Allergy and Anaphylaxis Network (NIAID/FAAN) [16]. All patients presenting with anaphylaxis to the emergency, outpatient, and inpatient departments during the 10-year study period were included. Patients under 16 years of age were classified as children [15].

Collected data included demographic characteristics, pre-existing medical conditions, underlying atopic diseases, history of allergies, triggers, clinical manifestations, time from allergen exposure to symptom onset, therapeutic interventions, clinical outcomes, and results of allergy evaluations.

Severe anaphylaxis was defined as the presence of at least one of the following: hypotension, cardiovascular collapse, respiratory failure or cyanosis, or loss of consciousness [15,17]. Hypotension was defined as systolic blood pressure < 70 mmHg in patients aged 1 month to 1 year; <70 + (age in years × 2) mmHg in patients aged >1 to 10 years; and <90 mmHg in patients aged >10 years. Cyanosis was considered if pulse oximetry saturation (SpO2) was less than 95%.

Recurrent anaphylaxis was defined as a subsequent episode meeting the NIAID/FAAN criteria. The total number of recurrent episodes per patient was documented within 10-year study period.

### 2.3. Candidate Predictors and Time Point of Prediction

Candidate variable predictors were selected using a pre-specification approach prior to analyzing the dataset, primarily based on findings from our previous study on risk factors for recurrent anaphylaxis using the same retrospective dataset [11], as well as clinical relevance and prior evidence from comprehensive literature reviews [5,7,9,10,11,12,13,14]. These predictors included baseline characteristics (e.g., age. sex, and history of food, insect, or drug allergy), comorbidities (e.g., asthma, atopic dermatitis, and cardiovascular diseases), triggers, presenting symptoms (e.g., urticaria, angioedema, cough, chest discomfort, and sensation of a lump in the throat), prescription of self-injectable epinephrine, and severity of the index anaphylactic episode.

These predictors were derived from clinical information available at the time of the index anaphylaxis episode (i.e., the time point of prediction) and were used to estimate the risk of future recurrent anaphylaxis.

### 2.4. Endpoint of Interest

The primary endpoint was the occurrence of recurrent anaphylaxis within the 10-year observation period. Prediction time horizon in this study was 10 years. Patients without recurrence were censored at the conclusion of the study period which was February 2021.

### 2.5. Sample Size Estimation

The minimum required sample size was determined using the pmsampsize method [18] for developing a binary clinical prediction model. Based on the predefined assumptions of evaluating 6 candidate predictors, an expected C-statistic of 0.80, and a recurrence prevalence of 0.132, an estimated minimum of 360 participants was required. This cohort size was projected to yield approximately 48 events of recurrent anaphylaxis.

### 2.6. Missing Data

Overall, missing data in this study were negligible (<5%). Missing values were limited to the variable ‘self-injectable epinephrine prescription upon discharge,’ which was unavailable for 5 patients (1.1%) who were referred to provincial hospitals due to medical insurance coverage. Given the minimal proportion of missing data, a complete-case analysis was performed without missing data imputation, as this was unlikely to compromise the integrity of the primary outcome.

### 2.7. Statistical Analysis and Model Development (Time-to-Event Modeling)

Statistical analyses were performed using Stata (Release 18; StataCorp LLC, College Station, TX, USA). Categorical variables were summarized as frequencies and percentages, while continuous variables were presented as medians with interquartile ranges (IQR). Comparisons of clinical candidate predictors between recurrent and non-recurrent groups were conducted using Fisher’s exact test. A two-sided *p*-value < 0.05 was considered statistically significant. Time-to-event analysis was applied to assess recurrence intervals and overall follow-up duration.

Based on the time-to-recurrence endpoint, candidate predictive risk factors were evaluated using univariable Cox proportional hazards regression model, with results reported as C-statistic for each parameter. A multivariable Cox proportional hazards regression model was then constructed to develop the prediction model. Adjusted hazard ratios (HRs) with 95% confidence intervals (CIs) were reported for all included predictors. Predictor selection was guided by a pre-specification approach prior to analysis of the dataset. To identify influential predictors and determine whether the removal or addition of predictors substantially changed the model, we performed a sensitivity analysis for comparison.

### 2.8. Derivation of the Point-Based Risk Score

The clinical predictors demonstrating statistical significance in the multivariable model, alongside parameters deemed clinically essential, were assigned individual item scores derived from their Cox regression β-coefficients. Specifically, each predictor was assigned a weight proportional to its β-coefficient. This was achieved by dividing the regression coefficient of each predictive factor by the lowest coefficient in the model and rounding the result to the nearest 0.5 to establish a standardized clinical risk score.

The cumulative risk score for each patient was then utilized to assess the model’s overall ability to predict recurrent anaphylactic episodes. To enhance clinical applicability, these total scores were further stratified into low-, moderate-, and high-risk categories. The predictive performance of each risk tier was evaluated based on the incidence of recurrence versus non-recurrence and quantified using positive likelihood ratios (LHR+) with 95% CI, with statistical significance defined as a *p*-value < 0.05.

### 2.9. Model Performance and Validation

To evaluate the predictive performance of the model, discriminative capacity was evaluated using the AuROC with 95% CIs [19]. Model calibration was assessed by comparing predicted and observed risks using calibration plots and summary indices, including the expected-to-observed (E:O) ratio, calibration-in-the-large (CITL), and the calibration slope [19].

Internal validation was subsequently performed using 500 bootstrap resamples to quantify and adjust for model optimism, ensuring the stability of the predictors [20]. Finally, to determine the clinical utility of the scoring system, a decision curve analysis (DCA) was conducted. This evaluated the net clinical benefit of the model across a range of clinically relevant threshold probabilities [21,22].

## 3. Results

### 3.1. Study Population and Outcomes

Medical records from Naresuan University Hospital between March 2011 and February 2021 were reviewed according to the inclusion criteria [11]. During the selection process, a total of 324 patients (381 episodes) were excluded from the analysis: 254 patients (286 episodes) had unretrievable records, 50 patients (75 episodes) had insufficient data, and 20 patients (20 episodes) did not meet the clinical diagnostic criteria for anaphylaxis [16].

The final cohort consisted of 381 patients with 439 anaphylaxis episodes over the 10-year study period. Among these, 58 episodes (13.2%) were classified as recurrent anaphylaxis. Of the 381 patients, 339 patients experienced a single episode, while 42 patients had multiple recurrent anaphylaxis, accounting for 58 recurrent events. Among patients with recurrence, 29 had one recurrence, 10 had 2 recurrences, and 3 had 3 recurrences [11].

### 3.2. Clinical Characteristics

Comparisons of candidate clinical predictors including baseline characteristics, underlying atopic diseases, and presenting symptoms between the recurrent and non-recurrent groups are summarized in Table 1. Missing data were negligible, with only five patients (1.1%) lacking information on self-injectable epinephrine prescription at discharge.

Compared with non-recurrent cases, patients with recurrent anaphylaxis had significantly higher proportions of a history of food allergy (56.9% vs. 27.3%, *p* < 0.001), a history of drug allergy (39.7% vs. 27.3%, *p* < 0.001), and a history of insect allergy (8.6% vs. 2.6%, *p* = 0.036). As for presenting symptoms, cough (8.6% vs. 2.9%, *p* = 0.047), chest discomfort (65.5% vs. 50.7%, *p* = 0.047), and a sensation of a lump in the throat (10.3% vs. 3.2%, *p* = 0.021) were significantly more common among patients with recurrence, whereas palpitations were less frequent (1.7% vs. 9.5%, *p* = 0.044).

Other variables, including sex, age, underlying atopic diseases, asthma, atopic dermatitis, underlying cardiovascular diseases, self-injectable epinephrine prescription, and severe anaphylaxis, were not significantly different between groups. In addition, the C-statistic values for individual variables, derived from univariable Cox proportional hazards regression, ranged from 0.50 to 0.66 (Table 1). The variable demonstrating the highest predictive ability was a history of drug allergy, with a C-statistic of 0.66 (95% CI 0.57–0.74).

### 3.3. Final Predictors and Score Derivation

Following the pre-specification process, six predictors were included in the final prediction model for recurrent anaphylaxis. Of these, five were derived from our previous study, which was designed to identify risk factors for recurrent anaphylaxis [11]. In that study, history of food allergy, history of insect allergy, history of drug allergy, chest discomfort, and severe anaphylaxis were identified as statistically significant risk factors in multivariable Cox regression analysis, with hazard ratios of 3.31 (95% CI: 1.50–7.29; *p* = 0.003), 4.96 (95% CI: 1.47–16.82; *p* = 0.010), 5.87 (95% CI: 2.64–13.07; *p* < 0.001), 2.43 (95% CI: 1.19–4.99; *p* = 0.015), and 2.29 (95% CI: 1.07–4.88; *p* = 0.033), respectively [11]. These five significant risk factors were subsequently incorporated into the final prediction model. The sixth predictor, asthma, was added before the analysis of the current dataset based on consistent evidence from multiple studies reporting asthma as a significant risk factor for recurrent anaphylaxis [5,9,12,13].

Among the six predictors included in the prediction model, multivariable Cox proportional hazards regression analysis (Table 2) demonstrated that three predictors achieved statistical significance: history of food allergy (HR 2.79; 95% CI: 1.47–5.29; *p* = 0.002; β = 1.03), history of insect allergy (HR 3.51; 95% CI: 1.22–10.08; *p* = 0.020; β = 1.26), and history of drug allergy (HR 4.17; 95% CI: 2.20–7.90; *p* < 0.001; β = 1.43). The remaining three predictors—underlying asthma, chest discomfort, and severe anaphylaxis—did not reach statistical significance in this model. Their corresponding estimates were as follows: asthma (HR 1.29; 95% CI: 0.49–3.38; *p* = 0.609; β = 0.25), chest discomfort (HR 1.86; 95% CI: 0.97–3.59; *p* = 0.063; β = 0.62), and severe anaphylaxis (HR 1.41; 95% CI: 0.74–2.69; *p* = 0.299; β = 0.34) (Table 2).

To evaluate the influence of the non-significant predictors and assess potential overfitting, we performed a sensitivity analysis comparing the full model (six predictors) with a reduced model containing only the three statistically significant predictors from the current data analysis: history of food allergy, insect allergy, and drug allergy. The full model consistently demonstrated a higher AuROC than the reduced model (0.773 [95% CI: 0.714–0.832] vs. 0.749 [95% CI: 0.686–0.812]), with an AuROC difference of 0.024. These findings suggest that the inclusion of asthma, chest discomfort, and severe anaphylaxis enhanced the model’s discriminative performance without substantially increasing the risk of overfitting. Furthermore, the bootstrap-corrected AuROC values were very similar to the corresponding apparent AuROC values for both the full model (0.773 vs. 0.773) and the reduced model (0.750 vs. 0.749). The corresponding optimism values were approximately 0.000 and −0.001, respectively, indicating minimal overfitting in both models. Detailed results are presented in the Supplementary Table S1.

Through the proportional weighting of the β-coefficients, risk scores were assigned as follows: history of drug allergy (5.5 points), history of insect allergy (5 points), history of food allergy (4 points), chest discomfort (2.5 points), severe anaphylaxis (1.5 points), and asthma (1 point). Total potential scores ranged from 0 to 19.5 points (Table 2).

### 3.4. Score Distribution and Risk Gradient

**Figure 1:**
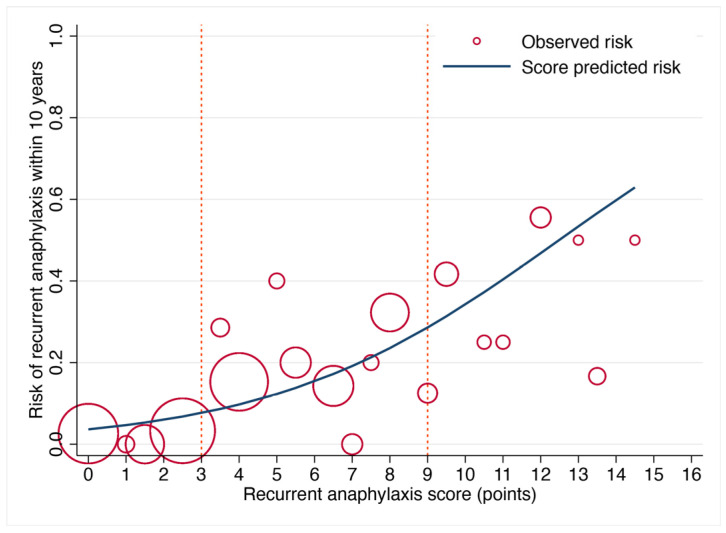
Risk curve for predicted versus observed recurrent anaphylaxis. The risk curve illustrates the correlation between the score-predicted risk (solid line) and the observed recurrence risk (hollow circles). The vertical dashed lines demarcate the three risk-group classifications (low, moderate, and high). The size of each circle is proportional to the number of patients at that specific score level.

The distribution of total scores derived from the predictive model differed significantly between patients with and without recurrent anaphylaxis, as illustrated in the risk curve (Figure 1). The curve demonstrates that the predicted risk of recurrences increased in correlation with higher total scores. Moreover, the observed risk at each score level aligned closely with the model’s predictions.

When stratified by total score, the rate of recurrent anaphylaxis increased across the risk levels (Table 3, Figure 1). In the risk curve (Figure 1), the three risk-group classifications are demarcated by vertical dashed lines. The predicted risk of recurrent anaphylaxis (*y*-axis) increased in a manner corresponding to the increase in our proposed clinical score (*x*-axis), with the circle size representing the proportion of patients at each data point. The total risk scores, ranging from 0 to 19.5 points, were categorized into 3 distinct tiers: low-risk (<3.0 points), moderate-risk (3.0–9.0 points), and high-risk (>9.0 points) (Table 3).

### 3.5. Model Performance: Discrimination and Calibration

**Figure 2:**
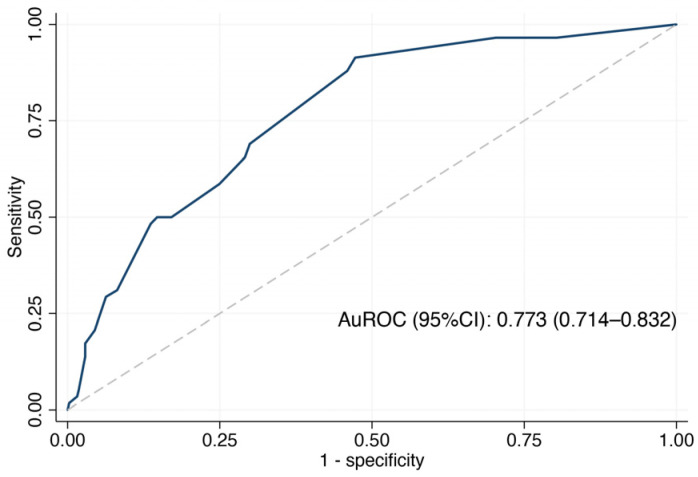
Receiver operating characteristic (ROC) curve of the predictive model. The area under the receiver operating characteristic (AuROC) curve, with a 95% confidence interval (CI), demonstrates the discriminative capacity of the prognostic prediction model in predicting recurrent anaphylaxis.

**Figure 3:**
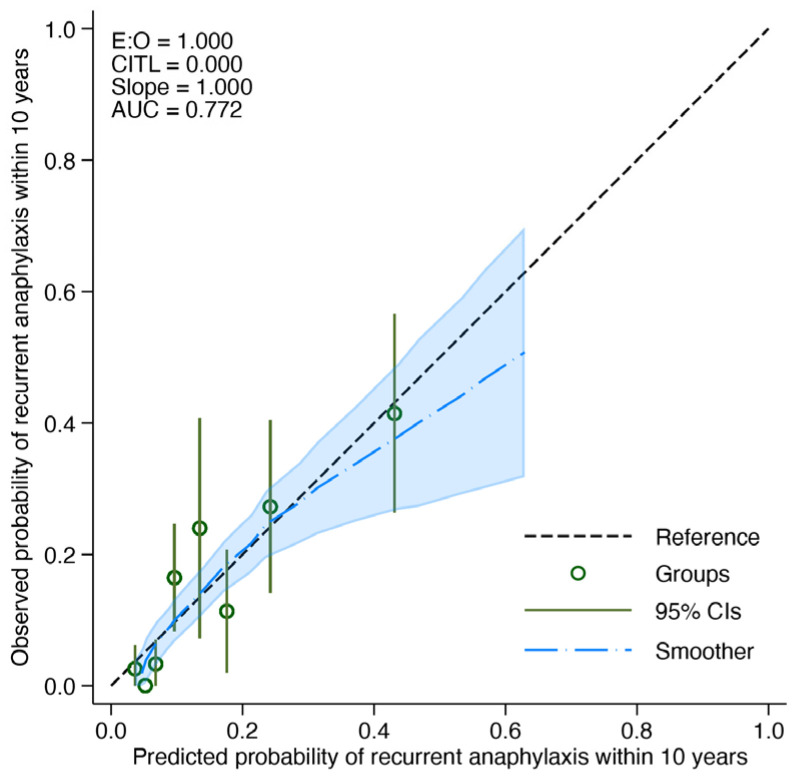
Calibration plot of the predicted versus observed risk of recurrent anaphylaxis. The plot illustrates the agreement between the score-predicted risk and the observed outcomes. CITL: calibration-in-the-large; E:O: expected-to-observed ratio.

The model demonstrated acceptable discriminative ability, with an AuROC of 0.773 (95% CI: 0.714–0.832) (Figure 2). Calibration plot analysis showed good agreement between predicted and observed risks of recurrent anaphylaxis (Figure 3). The model demonstrated an E:O ratio of 1.000, a CITL of 0.000, and a calibration slope of 1.000, indicating strong calibration performance.

### 3.6. Risk Categories and Positive Likelihood Ratios

Patients were stratified into three risk groups based on their total scores ranging from 0 to 19.5 points. The cut-off values for recurrent anaphylaxis risk stratification (<3.0, 3.0–9.0, and >9.0 points) were determined using positive likelihood ratio (LHR+) (Table 3). The low-risk group had an LHR+ of 0.16 (95% CI: 0.05–0.41; *p* < 0.001), indicating a significantly very low likelihood of recurrence. In contrast, the high-risk group had an LHR+ of 4.65 (95% CI: 2.19–9.63; *p* < 0.001), indicating a significantly high likelihood of recurrence. The moderate-risk group (scores 3.0–9.0) had an LHR+ of 1.52 (95% CI: 0.93–2.44; *p* = 0.088), which did not reach statistical significance. This finding suggests that patients in this group could not be clearly classified as either low or high risk and were therefore considered to have a moderate risk of recurrence.

### 3.7. Internal Validation

Internal validation using 500 bootstrap resamples confirmed the robustness of the model, yielding an optimism-adjusted AuROC of 0.773 (95% CI: 0.714–0.831), with an estimated optimism of approximately 0.000, indicating minimal overfitting. The shrinkage factor, representing an optimism-corrected estimate of calibration performance, was 1.02. These findings suggest good model stability and demonstrate that the model maintains strong calibration even after adjustment.

### 3.8. Decision Curve Analysis

**Figure 4:**
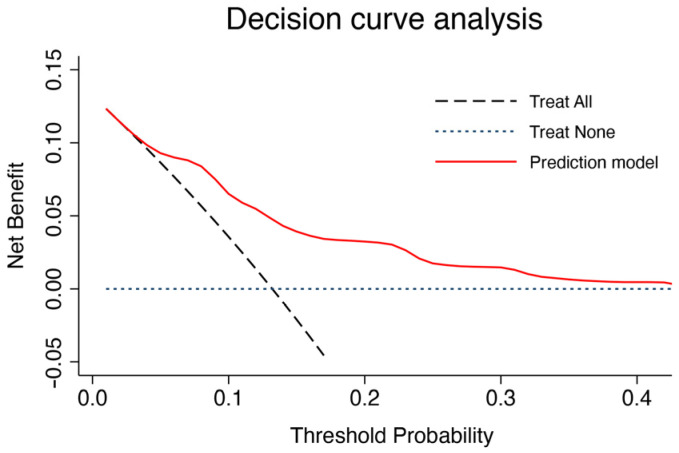
Decision curve analysis (DCA) of the predictive model. The decision curve analysis demonstrates the net clinical benefit of using the model to predict recurrent anaphylaxis across a range of clinically relevant threshold probabilities.

Decision curve analysis (DCA) demonstrated that the prediction model provided greater net benefit across a broad range of clinically relevant threshold probabilities compared with the conventional approaches of providing preventive care to all patients or to none (Figure 4). The model’s net benefit curve remained consistently higher than both reference strategies across most thresholds, indicating potential clinical utility. Additionally, the 10-year proportion of recurrent anaphylaxis in this study was 13.2%, which aligns with the threshold probability range where the model demonstrated the greatest net clinical benefit (Figure 4).

## 4. Discussion

Anaphylaxis is a severe and potentially life-threatening condition that often occurs unexpectedly. However, patients who experience an initial episode remain at risk for subsequent recurrent events. In this 10-year study, the proportion of recurrent anaphylaxis was 13.2% among anaphylaxis episodes, with a recurrence rate of 4.1 events per 100 person-years and a median time to recurrence of 9.9 months [11,15]. These findings are lower than those reported in some international studies, where recurrence rates have reached up to 54% and 90 episodes per 100 person-years [4,6], highlighting variability across populations and healthcare settings.

Prevention of recurrent anaphylaxis is critical, as early identification of high-risk patients may enable targeted monitoring and intervention. Previous studies have identified several risk factors for recurrence, including asthma [5,9,14], atopic dermatitis [7,9], food allergy [9,11,13], and a history of drug or insect allergy [11]. Additional factors such as allergic rhinitis, age < 18 years, and food-related triggers have also been associated with increased recurrence risk [5,9,10,12,13]. With respect to presenting symptoms, only a limited number of studies, including our previous report, have identified clinical manifestations such as cough, oral pruritus [7], chest discomfort, and severe anaphylactic symptoms [11] as significant predictors of recurrence.

Despite these findings, prediction models for recurrent anaphylaxis remain scarce. Such models are particularly valuable in clinical practice, as they provide a structured approach to risk stratification during emergency department encounters and early follow-up visits. Therefore, further investigation into the development of a predictive model was warranted, incorporating factors identified in both previous studies and our own analyses. To our knowledge, this is one of the first studies to develop a simple clinical prediction model for recurrent anaphylaxis intended for routine clinical use.

The final score-based model included six predictive factors: history of food allergy, history of insect allergy, history of drug allergy, asthma, chest discomfort, and severe anaphylaxis. Among these, three predictors—history of food allergy, insect allergy, and drug allergy—were statistically significant in the multivariable analysis, whereas the remaining variables did not reach statistical significance in this model. As previously described, these six predictors were selected through a pre-specification process. Five predictors—chest discomfort, severe anaphylaxis, history of food allergy, history of insect allergy, and history of drug allergy—were included based on their statistical significance as risk factors in our previous study on recurrent anaphylaxis. The final predictor, asthma, was additionally incorporated a priori because multiple studies have consistently identified it as a clinically relevant risk factor for recurrent anaphylaxis [5,9,12,13].

To evaluate the influence of the non-significant predictors and assess potential overfitting, we performed a sensitivity analysis comparing the final six-predictor model with a reduced model containing only the three statistically significant predictors. The full model with the inclusion of asthma, chest discomfort, and severe anaphylaxis demonstrated superior discriminative performance compared with the reduced model without evidence of substantial overfitting.The model demonstrated acceptable discriminative ability, with an AuROC of 0.773 (95% CI: 0.714–0.832), and showed good calibration, indicating close agreement between predicted and observed risks. Furthermore, internal validation using 500 bootstrap resamples yielded an optimism-adjusted AuROC of 0.773 (95% CI: 0.714–0.831), supporting the robustness of the model. Decision curve analysis demonstrated favorable clinical utility across a range of threshold probabilities, suggesting that use of this model may improve clinical decision making compared with non–model-based approaches.

The total risk score of the model ranged from 0 to 19.5 points and was stratified into three categories for recurrent anaphylaxis risk assessment. The cut-off thresholds (<3.0, 3.0–9.0, and >9.0 points) were established using positive likelihood ratio (LHR+). Evaluation of the model’s predictive performance showed that the low-risk group (<3.0 points) had a significantly very low probability of recurrence, whereas the high-risk group (>9.0 points) demonstrated a significantly high probability of recurrence.Implementation of this prediction model’s risk score during patient encounters may facilitate management planning and prevention of future anaphylactic events. In acute care settings, four predictors—history of food, insect, or drug allergy and underlying asthma—are routinely assessed and documented in medical records. In addition, the other two predictors, chest discomfort and symptoms of severe anaphylaxis, are also commonly evaluated during clinical assessment. To improve feasibility and consistency, acute care settings could implement structured checklists or electronic systems to document predictor variables and automatically calculate risk scores. Patients with anaphylaxis could then be managed according to their estimated recurrence risk scores to help prevent recurrence. However, the absence of external validation remains a potential barrier to implementation and may limit the model’s generalizability across different healthcare settings.

For patients classified as high risk (score > 9.0), management strategies should include closer observation, increased follow-up frequency, and a strong emphasis on investigation and prevention. Evaluation of this high-risk group should include allergy testing and assessment of comorbid allergic diseases. Preventive strategies may include allergen avoidance, optimal control of allergic rhinitis and asthma, carrying self-injectable epinephrine, and using an anaphylaxis identification card or wristband.

This study has several limitations. The retrospective observational cohort design resulted in some unretrievable or incomplete information, which may have influenced the interpretation of outcomes. In addition, the initial screening of medical records using ICD-10 codes specific to anaphylaxis may have excluded a small number of patients diagnosed under related codes, such as T78.3 (angioneurotic edema, laryngeal edema, Quincke edema, urticaria-larynx), T78.4 (allergy, unspecified), T78.9 (adverse effect, unspecified), and T80.9 (unspecified complication following infusion, transfusion, or therapeutic injection). Another limitation was the relatively small sample size, which may affect the model’s applicability to other populations and necessitates cautious interpretation. Moreover, as this is one of the first studies to develop a prediction model for recurrent anaphylaxis, there are no established simple rule-out criteria or standardized benchmarks against which to compare its performance. Further external validation is required before the model can be implemented in different healthcare settings. Nonetheless, this research represents the development of one of the first prediction models for recurrent anaphylaxis, offering a simple tool that uses readily available clinical predictors to estimate recurrence risk. This predictive model may assist healthcare personnel in improving medical decision making and providing more precise preventive and management strategies.

## 5. Conclusions

This research presents the development of a clinically applicable prediction model for estimating the risk of recurrent anaphylaxis. The model comprises six predictive risk factors: histories of food, insect, drug allergies, alongside asthma, chest discomfort, and severe anaphylaxis. These factors correspond to weighted scores of 4, 5, 5.5, 1, 2.5, and 1.5 points, respectively.

The cumulative risk score, ranging from 0 to 19.5 points, effectively classifies patients into three distinct risk tiers: low-risk (<3.0 points), moderate-risk (3.0–9.0 points), and high-risk (>9.0 points). Patients in the high-risk group demonstrated a significantly increased likelihood of recurrence. Overall, the model showed good statistical performance, with acceptable discrimination and calibration, supporting its potential utility in clinical practice.

This prediction model provides a simple tool to facilitate early risk stratification and guide preventive management strategies. Its use may enable clinicians to identify high-risk patients, implement targeted interventions, and improve clinical outcomes.

## Figures and Tables

**Table 1:** Candidate clinical predictors of recurrent versus non-recurrent anaphylaxis episodes over a 10-year study period, with corresponding C-statistic values.

Clinical Parameters	Missing Data n (%)	Recurrent Within 10 Years	Censored at 10 Years	*p*-Value	C-Statistic
		Numbers of Episodes (%) (n = 58)	Numbers of Episodes (%) (n = 381)		
Sex	0			0.199	0.58 (0.50–0.66)
Male		19 (32.8)	160 (42.0)		
Female		39 (67.2)	221 (58.0)		
Age	0			0.834	0.52 (0.49–0.60)
Age ≥ 16 years		50 (86.2)	332 (87.1)		
Age < 16 years		8 (13.8)	49 (12.9)		
History of food allergy	0	33 (56.9)	104 (27.3)	<0.001	0.63 (0.55–0.72)
History of drug allergy	0	23 (39.7)	104 (27.3)	<0.001	0.66 (0.57–0.74)
History of insect sting allergy	0	5 (8.6)	10 (2.6)	0.036	0.52 (0.49–0.56)
Underlying diseases	0				
Asthma		7 (12.1)	23 (6.0)	0.097	0.53 (0.50–0.59)
Atopic dermatitis	0	1 (1.7)	6 (1.6)	1.000	0.56 (0.50–0.64)
Cardiovascular disease	0	6 (10.3)	40 (10.5)	1.000	0.51 (0.49–0.56)
Trigger					
Food	0	31 (53.5)	184 (48.3)	0.484	0.53 (0.49–0.62)
Medication	0	10 (17.2)	51 (13.4)	0.418	0.54 (0.49–0.62)
Presenting symptoms					
Urticaria	0	25 (43.1)	169 (44.7)	0.888	0.50 (0.49–0.60)
Angioedema	0	17 (29.3)	145 (38.1)	0.243	0.54 (0.49–0.60)
Cough	0	5 (8.6)	11 (2.9)	0.047	0.53 (0.50–0.58)
Chest discomfort	0	38 (65.5)	193 (50.7)	0.047	0.56 (0.51–0.63)
Lump in the throat	0	6 (10.3)	12 (3.2)	0.021	0.52 (0.49–0.56)
Palpitation	0	1 (1.7)	36 (9.5)	0.044	0.53 (0.50–0.56)
Self-injectable epinephrine prescription upon discharge	5 (1.1)	11 (19.0)	39 (10.4)	0.074	0.53 (0.50–0.59)
Severe anaphylaxis	0	18 (31.0)	105 (27.6)	0.638	0.56 (0.50–0.65)

C-Statistic = concordance statistic; CI = confidence interval.

**Table 2:** Multivariable Cox proportional hazards regression analysis and assigned point-based risk scores for predicting recurrent anaphylaxis.

Predictive Risk Factors	HR	95% CI	*p*-Value	Coefficient	Score
History of food allergy	2.79	1.47–5.29	0.002	1.03	4
History of insect allergy	3.51	1.22–10.08	0.020	1.26	5
History of drug allergy	4.17	2.20–7.90	<0.001	1.43	5.5
Asthma	1.29	0.49–3.38	0.609	0.25	1
Chest discomfort	1.86	0.97–3.59	0.063	0.62	2.5
Severe anaphylaxis	1.41	0.74–2.69	0.299	0.34	1.5

HR = hazard ratio; CI = confidence interval; β = regression coefficient. Scores are derived from β coefficients of the multivariable Cox proportional hazards model and rounded to the nearest 0.5 for clinical applicability.

**Table 3:** Risk stratification categories for recurrent anaphylaxis and corresponding likelihood ratios.

Risk Categories	Score	Recurrence (n = 58)	Non- Recurrence (n = 381)	LHR+	95% CI	*p*-Value
Low	<3.0	5 (8.6)	201 (52.8)	0.16	0.05–0.41	<0.001
Moderate	3.0–9.0	36 (62.1)	156 (40.9)	1.52	0.93–2.44	0.088
High	>9.0	17 (29.3)	24 (6.3)	4.65	2.19–9.63	<0.001

Anaphylaxis episodes are presented as n (%). LHR+ = positive likelihood ratio; CI = confidence interval.

## Data Availability

Datasets generated and analyzed during the current study are available from the corresponding author on reasonable request.

## Supplementary Materials

The following supporting information can be downloaded at: https://www.mdpi.com/article/10.3390/jcm15113990/s1, Table S1: Sensitivity analysis comparing the performance of the full model (six predictors) with the reduced model (three statistically significant predictors).
