# Supplementary material for: Development of a Clinical Prediction Model for Recurrent Anaphylaxis
Source: J Clin Med. 2026 May 22;15(11):3990. doi: 10.3390/jcm15113990 (PMC13257516; doi:10.3390/jcm15113990)
Supplement: Supplementary file 1 [file jcm-15-03990-s001.zip › jcm-4284285-supplementary.pdf]

Table S1. Sensitivity analysis comparing the performance of the full model (six predictors) with the reduced model (three statistically significant predictors).

**Performance metric**	**Full model (6 predictors)** [a]	**Reduced model (3 predictors)** [b]
Discrimination		
Apparent AuROC (95% CI)	0.773 (0.714-0.832)	0.749 (0.686-0.812)
Bootstrap-corrected AuROC (95% CI) [c]	0.773 (0.714–0.831)	0.750 (0.686-0.818)
Optimism [d]	0	–0.001
Calibration		
Expected-to-observed ratio (E:O)	1.000	1.000
Calibration-in-the-large (CITL)	0.000	0.000
Calibration slope	1.000	1.000

Abbreviations: AuROC, area under the receiver operating characteristic curve; CI, confidence interval.

[a] Full model: history of food allergy, history of insect sting allergy, history of drug allergy, asthma, chest discomfort, and severe anaphylaxis.

[b] Reduced model: history of food allergy, history of insect sting allergy, and history of drug allergy (statistically significant predictors in the multivariable Cox proportional hazards regression in this prediction model).

[c] Bootstrap internal validation performed with 500 resampling iterations.

[d] Optimism = apparent AuROC − bootstrap-corrected AuROC.
